# Gram-negative infections: evolving treatments with expanding options

**DOI:** 10.4155/fsoa-2018-0071

**Published:** 2018-09-27

**Authors:** Maria Virginia Villegas, Sue Lyon

**Affiliations:** 1 Scientific Director Bacterial Resistance & Nosocomial Infections Research Area & Adjunct professor Universidad El Bosque, Bogota, Colombia; 2 Freelance Medical Writer & Editor, London, UK

**Keywords:** antibiotic resistance, antimicrobial, ECCMID, Enterobacteriaceae, Gram negative, multidrug resistance, stewardship

## Abstract

Antibiotic resistance was a major topic of interest for the nearly 13,000 physicians, microbiologists and scientists who attended European Congress of Clinical Microbiology & Infectious Diseases (ECCMID) 2018. Much discussion centered round the potential benefits of novel antibiotics that are either already approved or under investigation in the treatment of infections caused by resistant Gram-negative pathogens. There was also general acceptance of the importance of ensuring that antibiotic stewardship is implemented in every ward throughout every hospital to ensure that these novel drugs are used appropriately and to combat the development of resistance.

Gram-negative bacteria such as *Klebsiella*, *Acinetobacter* and *Pseudomonas* cause some of the most serious infections and are increasingly resistant to multiple drugs and, in some cases, most available antibiotics. Antimicrobial resistance is currently responsible for an estimated 700,000 deaths each year throughout the world, but if no action is taken, it is estimated that by 2050 there will be over 10 million deaths at a cumulative global economic cost of US$100 trillion [1].

Dr Rafael Cantón (Madrid, Spain) commented that, although surveillance studies show a global increase in extended-spectrum beta-lactamase (ESBL) and carbapenemase-producing Enterobacteriaceae (CPE) and multidrug resistant (MDR) *Pseudomonas aeruginosa*, there is wide intercountry variation in the levels of antimicrobial resistance. Within Europe, for example, resistance is generally higher in isolates of *Klebsiella pneumoniae*, *Acinetobacter spp.* and *P. aeruginosa* in southern and eastern countries than in northern countries [2].

Susceptibility to antibiotics may vary according to the type of bacteria and the site of infection. Resistance is more frequent, for example, in *P. aeruginosa* isolates from pneumonia than from bloodstream infections [2]. In addition, resistance varies within hospitals and may be higher in medical wards than in the intensive care unit (ICU). Levels of resistance may also fluctuate, indicating the importance of long-term and regular surveillance throughout each institution [3].

A study presented during ECCMID 2018 highlighted that, due to a patient's severity of infection and risk factors, institutions may face a double threat from antibiotic resistance. In 287 US hospitals, there was a moderate, but significant, correlation between carbapenem nonsusceptible *P. aeruginosa* and Enterobacteriaceae. Of hospitals in the highest quartile for carbapenem nonsusceptible Enterobacteriaceae, 46.2% were in the highest quartile for carbapenem nonsusceptible *P. aeruginosa* (p < 0.0001). Correlation was significant overall when stratified by hospital teaching status, size and infection onset [4].

The clinical consequences of antibiotic resistance among Gram-negative bacteria are clear, concluded Dr Cantón. Mortality is significantly increased in carbapenem-resistant infections as well as in MDR *P. aeruginosa*, especially causing nosocomial pneumonia [5]. Even when MIC is in the susceptible range, treatment failure is more likely, and mortality rises if a strain has a high MIC compared with a low MIC [6].

## Antimicrobial selection in at-risk patients

As resistance rises among Gram-negative pathogens, there are fewer treatment options and less likelihood of adequate empirical therapy, agreed Dr Yoav Golan (MA, USA). The patient mortality progressively increases in association with the administration of effective antimicrobial therapy [7]; however, not only mortality is important but also toxicity linked to the use of last resort antibiotics such as colistin, which may have particularly adverse consequences for some severely ill patients [8].

Dr Golan emphasized the importance of identifying patients who might benefit from the novel antibiotics that are now, or will shortly be, available. On the other hand, there are numerous risk factors for infections by resistant Gram-negative pathogens, and they can be summarized as hospitalization in long-term care and hemodialysis centers, long-antibiotic exposure and host factors (e.g., age, immune status, diabetes, chronic wounds, carrier status and recent infections). Although these factors will not identify the pathogen, the patient's medical history can be suggestive; for example, previous exposure to carbapenems and fluoroquinolones is a risk factor for drug-resistant *P. aeruginosa* [9].

Ultimately, the choice of adequate antibiotic therapy must be guided by up-to-date surveillance information. A study presented during ECCMID 2018 reported that, despite combining an aminoglycoside or fluoroquinolone with a beta-lactam antibiotic, *P. aeruginosa* susceptibility did not reach guideline-recommended target rates in almost all regimens surveyed. The researchers recommended evaluating local susceptibilities and newer antipseudomonal agents when managing patients with suspected or confirmed *P. aeruginosa* pneumonia or bacteraemia in both ICU and non-ICU settings [10].

## Clinical use of new Gram-negative antimicrobials

Ceftazidime–avibactam and ceftolozane–tazobactam currently are the only two novel antibiotics approved in both Europe and the USA. Dr Andrew Shoor (Washington DC, USA) reminded delegates that data required by regulators when approving new drugs are not necessarily relevant in routine clinic practice. The precise clinical application of novel Gram-negative antimicrobials is therefore currently unclear, but ‘real world’ case reports can be helpful in indicating how colleagues are using these new options.

A large US study presented during ECCMID 2018 reported that ceftolozane–tazobactam was highly effective in treating complex urinary tract infections (UTI) or complex intra-abdominal infections in 103 patients. Median length of stay from first dose of ceftolozane–tazobactam was 6 days (IQR: 4–15), and 83% of patients were discharged within 30 days of the last course of Gram-negative treatment. Mortality was 14%, and 17 patients had an infection-related readmission within 30 days [11].

Dr David Nicolau (CT, USA) was especially concerned about managing MDR *P. aeruginosa*. In general, practice has been to optimize the dose and switch to continuous and prolonged infusion to increase antimicrobial efficacy. However, in the context of rising MICs it has become important in clinical microbiology to understand precise mechanisms of resistance and the profile of the phenotype of resistant organisms in each institution [12].

Given the mortality associated with MDR *P. aeruginosa*, Dr Nicolau was encouraged by the activity of ceftolozane-tazobactam against this bacterium. Ceftolozane–tazobactam is stable against common *P. aeruginosa* resistance mechanisms, including loss of outer membrane porin (OprD), chromosomal AmpC and upregulation of efflux pumps. Isolates resistant to other antipseudomonal β-lactams may be still susceptible to ceftolozane–tazobactam, though cross-resistance may occur [12].

Turning to carbapenem-resistant *Enterobacteriaceae*, Dr Nicolau highlighted potential advantages of ceftazidime–avibactam compared with colistin. Because of colistin's poor pulmonary penetration, monotherapy is generally avoided in preference to combination therapy with colistin, meropenem and tigecycline. Compared with a colistin-based regimen, ceftazidime–avibactam has been shown to be less toxic and to reduce infection-related morbidity and mortality [13].

Currently under investigation, imipenem-relebactam also offers a promising alternative to colistin, continued Dr Nicolau. Relebactam restores the activity of imipenem against resistant Gram-negative bacteria, and it is active *in vitro* against *Escherichia coli*, *K. pneumoniae* and *Enterobacter spp*, including KPC-producing isolates in *P. aeruginosa* and Enterobacteriaceae [14]. In the Phase III RESTORE-IMI 1 study, presented during ECCMID 2018, *in vitro* activity translated at Day 28 into a higher clinical response and lower all-cause mortality in the patients with infections caused by imipenem-nonsusceptible pathogens [15].

## Antimicrobial stewardship: strategic approaches

Antibiotic stewardship is a key to optimizing the use of existing and novel antibiotics. According to Professor MV Villegas (Bogota, Colombia); there are ‘five golden rules’ for creating an antimicrobial Guideline, which is one of the first steps in any antimicrobial stewardship program. The first rule is to know the hospital local epidemiology; the second is to investigate prevalent circulating mechanisms of resistance in each institution; the third is understanding the concept of selective pressure; the fourth is to implement patient and disease stratification as well as to incorporate risk factors for MDR bacteria, and finally to ensure de-escalation of treatment, which means reducing duration, dose, time and broad-spectrum to narrow spectrum of the antibiotic selected once the cultures are available and the patient is stable.

Selective pressure is an especially important concept, continued Professor Villegas. As such, the broadest spectrum of antibiotics, like carbapenems, should be used only when absolutely necessary. Selection of resistant *P. aeruginosa* during imipenem therapy is substantially more frequent than selection of ciprofloxacin-, piperacillin- and ceftazidime-resistant mutants, and increases the risk of intestinal colonization by resistant clones [16,17]. She added that in her hospital, adherence to local carbapenem-sparing antimicrobial guidelines reduced the risk of severe sepsis and other clinical complications in patients with complicated UTI [18].

The patient and disease stratification involves assessment of the risk of failure according to the severity of the infection, helping the physician determine whether initial treatment should be with a narrow- or broad-spectrum antibiotic. Professor Villegas reminded delegates that the implications for the patient are very different if, for example, inappropriate therapy is given for cystitis versus pyelonephritis. Concentration of the drug to the site of infection is also a key consideration. Despite resistance, the generally high concentration of an antibiotic in a UTI may result in clinical success when the same antibiotic would be ineffective in a bloodstream infection.

It is also essential to measure and report outcomes of antimicrobial stewardship to ensure continuing support for the program within an institution. Professor Villegas cited a study comparing outcomes of healthcare-associated infections before and after implementation of an antibiotic stewardship program at four acute care hospitals in two Colombian cities. Following implementation, adherence to guideline-recommended therapy rose from 9 to 45% and the rate of treatment de-escalation increased from 8 to 92%. Clinical improvement was 10-times more likely when antibiotic therapy was chosen according to the program than when patients were not treated under the program. Following introduction of the program, length of stay fell (10.8 vs 14 days before the program) and total infection costs were lower (US$3307 vs US$4655; p < 0.001) [19].

Professor Villegas advised delegates that any antibiotic stewardship program should be tailored to the institution. One of the hospitals in the study had only one infectious disease physician, so nurses were trained to implement the program on the wards. This raised some concern among prescribing physicians, but 6 months after implementation when the benefits for patients had become clear, doctors and nurses were working together to determine choice of antibiotic. Professor Villegas concluded that clinical champions play a crucial role in delivering a change in institutional culture that ensures continuing adherence to antibiotic stewardship in every ward in the hospital.

## Conclusion

When treating serious Gram-negative infections, timely use of the right antibiotic is critical to patient survival, but choice of empirical therapy is complicated by antibiotic resistance. Awareness of local epidemiology, effective teamwork and stringent antibiotic stewardship will be mandatory to ensure that novel antibiotics are used appropriately.
